# Ablation outcome of irreversible electroporation on potato monitored by impedance spectrum under multi-electrode system

**DOI:** 10.1186/s12938-018-0562-9

**Published:** 2018-09-20

**Authors:** Yajun Zhao, Hongmei Liu, Suyashree P. Bhonsle, Yilin Wang, Rafael V. Davalos, Chenguo Yao

**Affiliations:** 1 State Key Laboratory of Power Transmission Equipment and System Security and New Technology, School of Electrical Engineering, Chongqing University, No. 174 Shazhengjie, Shapingba District, Chongqing, 400044 China; 2 Department of Biomedical Engineering and Mechanics, Virginia Tech, 329 ICTAS Stanger St (0298), Blacksburg, VA 24061 USA

**Keywords:** Electroporation assessment, Irreversible electroporation, Bioimpedance, Equivalent circuit model, Ablation size, Tumor therapy

## Abstract

**Background:**

Irreversible electroporation (IRE) therapy relies on pulsed electric fields to non-thermally ablate cancerous tissue. Methods for evaluating IRE ablation in situ are critical to assessing treatment outcome. Analyzing changes in tissue impedance caused by electroporation has been proposed as a method for quantifying IRE ablation. In this paper, we assess the hypothesis that irreversible electroporation ablation outcome can be monitored using the impedance change measured by the electrode pairs not in use, getting more information about the ablation size in different directions.

**Methods:**

Using a square four-electrode configuration, the two diagonal electrodes were used to electroporate potato tissue. Next, the impedance changes, before and after treatment, were measured from different electrode pairs and the impedance information was extracted by fitting the data to an equivalent circuit model. Finally, we correlated the change of impedance from various electrode pairs to the ablation geometry through the use of fitted functions; then these functions were used to predict the ablation size and compared to the numerical simulation results.

**Results:**

The change in impedance from the electrodes used to apply pulses is larger and has higher deviation than the other electrode pairs. The ablation size and the change in resistance in the circuit model correlate with various linear functions. The coefficients of determination for the three functions are 0.8121, 0.8188 and 0.8691, respectively, showing satisfactory agreement. The functions can well predict the ablation size under different pulse numbers, and in some directions it did even better than the numerical simulation method, which used different electric field thresholds for different pulse numbers.

**Conclusions:**

The relative change in tissue impedance measured from the non-energized electrodes can be used to assess ablation size during treatment with IRE according to linear functions.

## Background

Electroporation is a phenomenon that causes an increase in the plasma membrane permeability due to the application of short (~ 100 μs), high voltage pulsed electric fields (PEFs) (~ 1000 V/cm) across the cell [1, 2]. Depending on the nature of the applied PEFs and pulse parameters, the pores can either induce a reversible or irreversible effect on the biological cell [3]. Electroporation is ‘reversible’ when the pores reseal after pulse delivery and cell viability is maintained. Conversely, electroporation is ‘irreversible’ when the cell dies because of loss of homeostasis even after the pulse application is terminated [4]. Irreversible electroporation (IRE) has been used to ablate undesirable tissues like tumors [5–7] and for other applications such as food processing [8]. Regarding the tumor therapy of IRE, assessment of ablation outcome is critical for successful treatment. By evaluating the ablation zone after treatment, one would know when to stop treating to avoid over-treatment or under-treatment. Prior studies have shown that changes in tissue properties can be used to detect the ablation results, such as the variation in conductivity and resistance [9–11], electrical impedance tomography (EIT) [12, 13] and magnetic resonance electrical impedance tomography (MREIT) [14, 15]. Typically, IRE treatments use 2–6 needle electrodes inserted in or around the tumor to apply the PEFs. During each pulse delivering process, only two electrodes are employed to apply high voltage treatment pulses while the other electrodes can be used to measure tissue property changes. However, few studies have investigated the information obtained from the non-pulse delivering electrodes, while the tissue property changes measured by the treatment electrodes has been reported in some papers [10, 17, 18].

In this study, we propose a technique to characterize the IRE ablation zone for a four-needle setup. Both the treatment electrodes and the dormant electrodes were used to measure tissue impedance changes. This process was conducted on potato tuber tissue, a model commonly used for electroporation studies since the electroporated area becomes dark, which makes it is easy to define the ablation boundary [16–22]. First, the two diagonal electrodes in a four-needle setup were used to apply electroporation-inducing pulses to potato tissues. After treatment, the impedance between different pairs of electrodes was measured and compared to the values before treatment. A circuit model was then used to extract the equivalent element value of every circuit element, qualifying the relative change of the impedance. Finally, three functions were used to correlate the relative impedance change and the ablation size, and these functions were also employed to predict the ablation size caused by pulses with different pulse parameters. The method proposed in this work could successfully characterize the ablation zones during IRE treatment using a four-needle configuration and should be translated to a six-needle configuration in the future.

## Methods

### Multi-electrode experiment

**Fig. 1:**
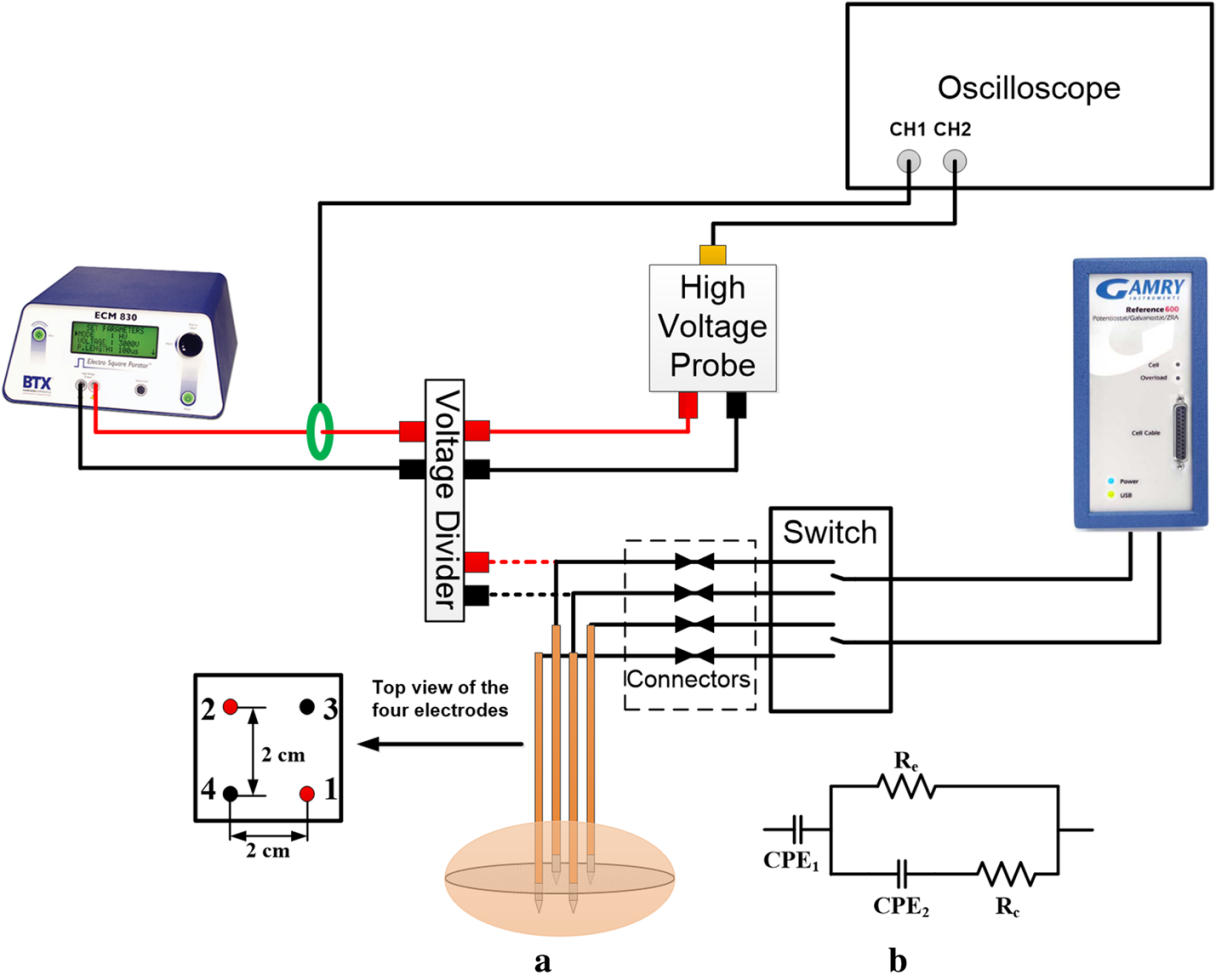
Schematic of the experimental setup (**a**) and the equivalent circuit model (**b**). The dashed line in (**a**) indicates that these two lines are shared with the two lines connected to Gamry, and the four connectors should be disconnected when applying high voltage pulses. The shadow in the potato is the mid-plane of the ablation zone that was cut

To compare the impedance spectrum from different electrode pairs, a construction of four electrodes was established to treat the potato. Figure 1a shows the schematic of the experimental setup. The pulses were generated by an ECM 830 Square Wave Electroporation System (Harvard Apparatus, Holliston, MA, USA). An oscilloscope (DPO 2012, Tektronix, Inc., Beaverton, OR) with a high voltage probe (BTX Enhancer™ High Voltage Probe, Harvard Apparatus, Holliston, MA) and current sensor (Pearson Electronics Inc., Palo Alto, USA) was used to record the voltage and current. Four 1.15 mm diameter monopolar electrodes (AngioDynamics, Inc., Latham, NY) were attached via laser-etched electrode holders to create a square-shaped array with a separation of 2 cm and the exposure lengths of the electrodes were all set to 1 cm. All four electrodes were inserted in the whole potato tuber without prior slicing. Two electrodes, 1 and 2, at the diagonal, were chosen to apply treatment pulses. After each treatment, four needles were used to mark the electrode positions for slicing 48 h after treatment. Before and after treatment, an impedance analyzer (Gamry, Warminster, PA, USA) was used to measure the impedance spectrums between electrodes 1–2, 1–4, 3–2, and 3–4. The frequency range was chosen from 1 Hz to 1 MHz at ten points per decade. The switchboard was custom built to realize the impedance measurement between different electrode pairs. During treatment, the impedance analyzer was disconnected to avoid breaking the equipment due to the high voltage. To guarantee this disconnection, four connectors with visible contacts were added. Therefore, the electrodes were connected or disconnected to the impedance analyzer manually, and the time for connection for different groups was similar and within 10 s. In other words, all the impedance spectrum measurements from different electrode pairs were started within 10 s after treatment. The pulse parameters used in this study are shown in Table 1. Recently, a study showed that the best time to slice the potato sample is 48 h after treatment if the potato is treated as a whole as it is here [18], so the samples were sliced 48 h after applying pulses. As shown in Fig. 1b, the shadow in the potato tuber is the plane in which it was cut. Before slicing, the samples were stored at room temperature.

**Table 1:** Pulse parameters used in four needle electrodes

Group	Pulse amplitude (V)	Pulse width (μs)	Pulse number	Interpulse delay (s)
1	500	100	30	1
2	500	100	60	1
3	500	100	90	1
4	750	100	30	1
5	750	100	60	1
6	750	100	90	1
7	1000	100	30	1
8	1000	100	60	1
9	1000	100	90	1
10^a^	800	100	30	1
11^a^	800	100	60	1
12^a^	800	100	90	1

^a^These pulse parameters were used to verify the methods used to monitor the ablation outcome proposed in this study

### Equivalent circuit model

To extract more information from the impedance spectrum, an equivalent circuit model (ECM) was used to fit the impedance data. This process was done in Gamry Echem Analyst software (Gamry, Warminster, PA, USA). The typical ECM for tissue is the Cole–Cole model [23], shown in Fig. 1b. The double layer effect at the interface between electrodes and tissue is quantified by a constant phase element (CPE) [19]. The impedance of CPE is given in Eq. (1):

1 $$Z_{\text{cpe}} = \frac{1}{{Q(j\omega )^{n} }}$$

Here, *ω* is the angular frequency, *j* is the unit imaginary number, *Q* and *n* are the empirical parameters.

The polarization of the cell membrane in tissue-scale is described by another CPE while in cell level it is equivalent to a capacitor. At the cell level, the membrane only has one single time constant; however, at the tissue level, the time constant is space distributed, and this is reflected by the coefficient *n* in CPE [24]. The Cole–Cole model also takes into account the intracellular and extracellular current pathways (*R*_c_ and *R*_e_ in the ECM). After electroporation, there will be a new pathway for the current, leading to a new resistance in parallel with *R*_e_. In the present study, this new resistance is combined to construct a new *R*_e_ after electroporation. Therefore, at the tissue level, the *R*_e_ in the ECM not only represents the pure extracellular resistance but also reflects the degree of electroporation. To eliminate the difference of the property from various potatoes, *R*_rel_ was defined to quantify the variation of *R*_e_ and can be easily used in the subsequent data analysis:

2 $$R_{rel} = \frac{{R_{e0} }}{{R_{e1} }}$$

Here, *R*_e0_ is the equivalent extracellular resistance before treatment, and *R*_e1_ is the updated equivalent extracellular resistance, consisting of the original *R*_e0_ and the new pathway for current because of electroporation.

### Quantization of ablation size

**Fig. 2:**
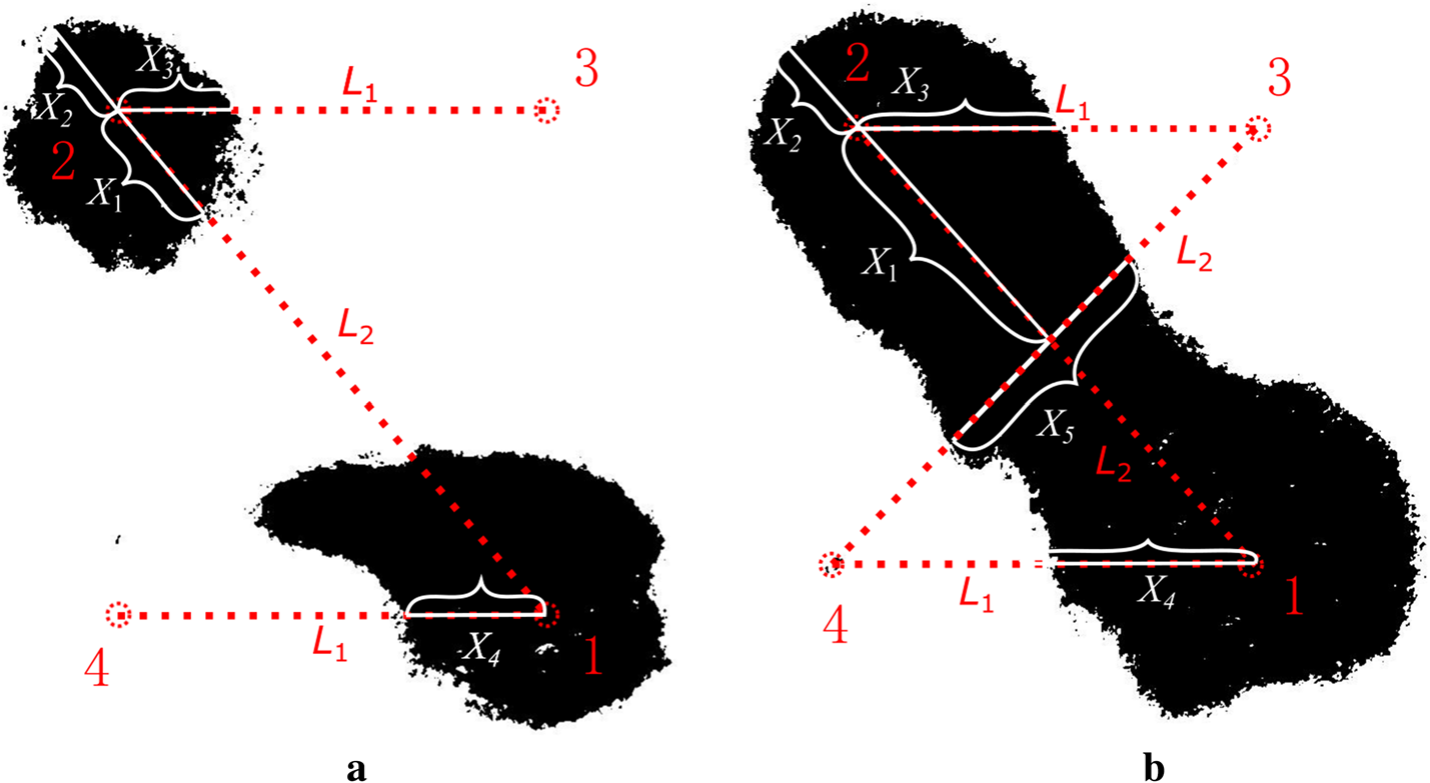
Sketch of the square edge, diagonal and length of the ablation area in different directions. **a** Ablation zone is not connected—treated by 30, 500 V pulses (group 1). **b** Ablation zone is connected—treated by 90, 500 V pulses (group 3)

After slicing the samples, the images of the potatoes were converted to 8-bit grayscale images to enhance the contrast (Fig. 2) as previously described [17]. If we connect any two electrodes with a straight line, there will be some cross-points between the line and the ablation area, given in Fig. 2. The position of these points was measured using ImageJ (National institutes of Health, Bethesda, MD, USA). Most importantly, the location of these points helped us estimate the ablation size and the location. These lengths in the specific directions were used to quantify the ablation size and defined as *X*_1_, *X*_2_, *X*_3_, *X*_4_ and *X*_*5*_. Moreover, to make variables more universal, we transferred them into dimensionless variables (*k*_1_, *k*_2_, *k*_3_, *k*_4_, *k*_5_) by normalizing them to the length of the connecting line between the electrodes in associated direction as the Eq. (3):

3 $$\begin{aligned} k_{1} = X_{1} /L_{2} \hfill \\ k_{2} = X_{2} /L_{2} \hfill \\ k_{3} = X_{3} /L_{1} \hfill \\ k_{4} = X_{4} /L_{1} \hfill \\ k_{5} = X_{5} /L_{2} \hfill \\ \end{aligned}$$

Here, *L*_1_ and *L*_2_ are the lengths of the square edge and diagonal, respectively. *X*_1_–*X*_5_ are the lengths of the ablation area in a specific direction, shown in Fig. 2. To cover every possible ablation outcome that can be reflected by *k*_1_–*k*_5_, two main ablation phenomena were included: the ablation zone not connected (Fig. 2a) and ablation zone connected (Fig. 2b). In the condition of Fig. 2a, there is no *X*_5_ (or *k*_5_) but *X*_1_ (or *k*_1_) is meaningful for ablation assessment. In contrast, *X*_5_ is meaningful for ablation size evaluation but *X*_1_ will keep constant in the condition of Fig. 2b. In Fig. 2, the ablations in the two figures were created by pulses with different pulse protocols to illustrate those two conditions. Figure 2a shows the ablation treated by 30, 500 V pulses (group 1) while (b) was treated by 90, 500 V pulses (group 3).

The variation in the ablation area, which is reflected by *k*_1_, *k*_2_, *k*_3_, *k*_4_, *k*_5_ to some degree, resulted in differences on the impedance spectrum. Therefore, there should be a relationship between *R*_rel_ (reflect the change of impedance) and *k*_1_–*k*_5_ (reflect the ablation area). Three functions (for different directions) were employed to describe the relationships by fitting the experimental data to the corresponding mathematic model. Considering the symmetry of ablation zone, in theory, *k*_3_ and *k*_4_ are merged into one variable *k*_3,4_ when the date fitting is done.

### Verification of the methods

To verify whether the functions proposed here can be used to predict the ablation size based on the change of the impedance, different pulse protocols (group 10–12 in Table 1) were used to treat the potato tissue, and the impedance spectrums from different electrode pairs before and after treatment were recorded and transferred into *R*_rel_ by circuit model and Eq. (2). The three functions proposed before were used to calculate the ablation size based on the acquired relative change of *R*_e_. The results predicted by the functions were then compared to the real measurement and the errors were calculated:

4 $$Error = \frac{{|X_{m} - X_{f} |}}{{X_{m} }}\times100\%$$

Here, *X*_m_ is the length of the ablation size in a specific direction measured experimentally and *X*_f_ is the length calculated by the functions.

Oftentimes, numerical simulations are used to estimate the ablation size ahead of time for a given pulse protocol. Here, the numerical simulation was also done and the results of the simulation were compared to the experimental results; the error was then calculated by Eq. (4). From the error of the method we proposed in this study and the error of the commonly used simulation method, we can evaluate the effectiveness of the method proposed here. For the simulation, the electrode configuration and the pulse amplitude were the same as the experiment. The dynamic conductivity in [16] was included in the simulation model.

## Results

### Comparison of the impedance spectrum

**Fig. 3:**
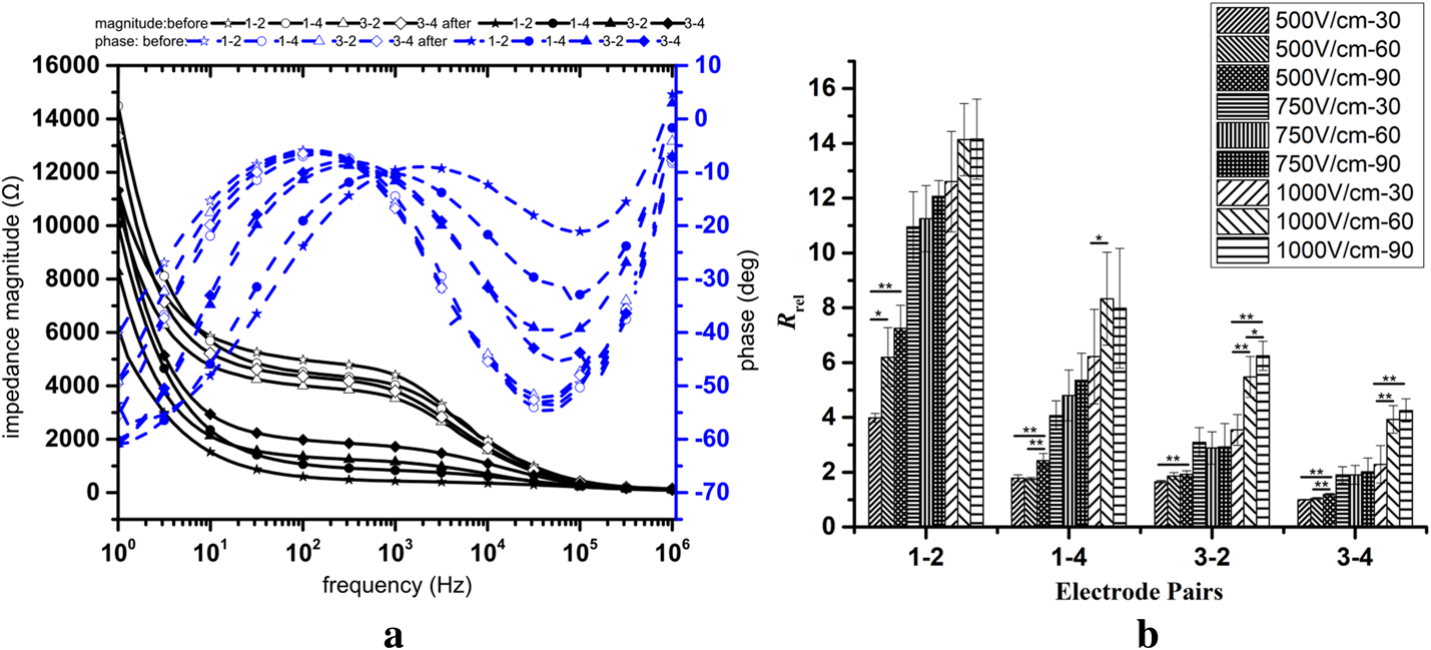
Impedance spectrum (**a**) and *R*_rel_ between different electrode pairs. In **a**, the applied pulse is with group 4 parameters (pulse amplitude: 750 V, pulse number: 30) in Table 1. In **b**, 500 V-30 means the magnitude of the electric field is 500 V and the pulse number is 30 (group 1 in Table 1). (**p < 0.01, *p < 0.05)

The typical impedance spectrums of potatoes from different electrode pairs, before and after treatment, are shown in Fig. 3a. Before treatment, the ratio of the impedance spectrums from electrodes 1–2 and 3–4 should be √2 due to differences in the distance between different electrode pairs, and the impedance between pairs 1–2 and 3–4, 1–4 and 3–2 should be the same. However, in reality, the results always have some error because the potato is not absolutely homogenous, and there are deviations in the electrode insertion process. After treatment, the impedance spectrum from 1–2 changed the most while 3–4 changed the least, as expected. The change in impedance from 1–4 and 3–2 should be the same because of geometrical symmetry; however, in reality, like the impedance measurement before treatment, it is always a little different from the theoretical value. After treatment, electrolysis at the anode and cathode may also have different effects on the impedance because we used monopolar pulses here [25].

Figure 3b shows *R*_rel_ among different electrode pairs after fitting the impedance data to the ECM shown in Fig. 1b. The results are consistent with Fig. 3a; the change of *R*_rel_ from 1–2 is maximum while *R*_rel_ from 3–4 has the minimal change. The variations of *R*_rel_ between 1–4 and 3–2 are similar. When treated by pulses with the group 1 parameters (30, 500 V), *R*_rel_ from 3–4 is close to unity, which means that there is no impedance change from 3–4 before and after treatment.

The one-sided Student’s *t* test with unequal variances was performed to analyze the statistical significance between groups treated by different pulse numbers with constant pulse amplitude and the results are shown in Fig. 3b. The results showed that the change of the resistance from 1–2 has a larger deviation than other electrode pairs. Only two statistical differences from electrode pair 1–2 were observed while three from 1–4 and four from 3–2 and 3–4 were found.

### Correlation between the impedance change and the ablation zone

**Fig. 4:**
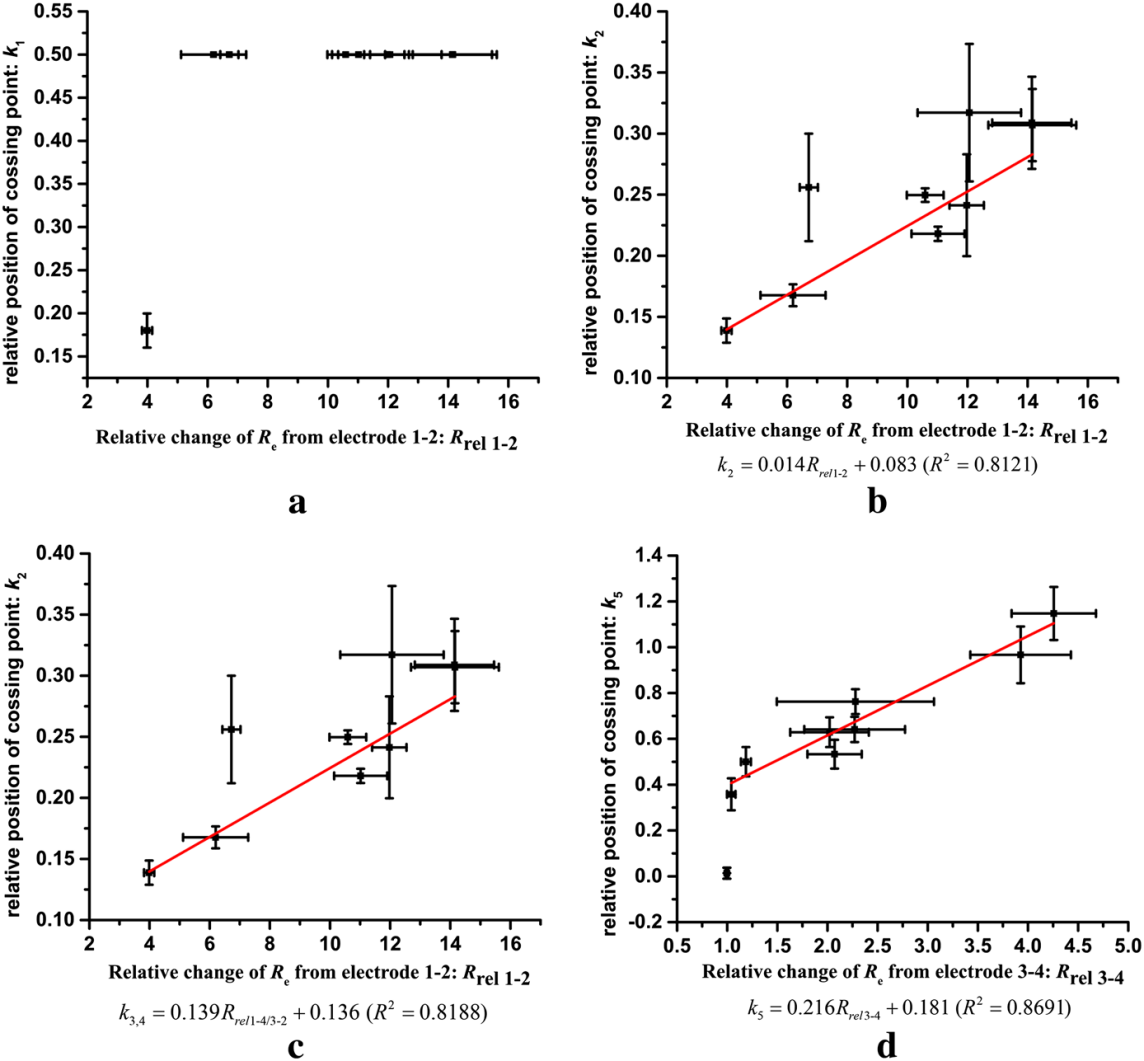
Position of the cross-point and the value of *R*_rel_. The data shown in the figure is the mean (central point) ± standard deviation (bar). The best fitting functions to describe the relationship of *R*_rel_ and the position of the ablation zone (*k*_2_–*k*_5_) are shown with the coefficient of determination in each figure. **a** The relationship between *k*_1_ and *R*_rel 1–2_ from electrode pair 1–2, **b** the relationship between *k*_2_ and *R*_rel 1–2_ from electrode pair 1–2, **c** the relationship between *k*_3,4_ and *R*_rel__1–4/3–2_ from electrode pair 1–4 and 3–2, **d** the relationship between *k*_5_ and *R*_rel 3–4_ from electrode pair 3–4

After slicing and converting the image of the ablation zone, ImageJ was used to determine the relative position of each cross-point, and the results are shown in Fig. 4. Figure 4a shows the change of *k*_1_ versus *R*_rel 1–2_ from 1–2. When the two ablation zones around electrode 1 and 2 are connected, *k*_1_ = 0.5. Figure 4b shows the values of *k*_2_ and *R*_rel 1–2_ from 1–2. A linear function was used to fit the data, and the coefficient of determination (R^2^) is given. Figure 4c shows the results of *k*_3_, *k*_4_ and the *R*_rel 1–4/3–2_ from 1–4 and 3–2. Due to theoretical symmetry, the values of *k*_3_ and *k*_4_ are merged into one parameter, *k*_3,4_ in Fig. 4c. Also, the fitted function and the *R*^2^ value are given. Figure 4d gives the values of *k*_5_ and *R*_rel 3–4_ from 3–4. After the two ablation zones around the electrodes are connected (*k*_5_ > 0), a linear function can accurately describe the relationship between *k*_5_ and *R*_rel 3–4_ from 3–4. The expression of the function and *R*^2^ are shown below in Fig. 4d.

### Comparison between the method in this study and the numerical simulation

Table 2 shows the results of the ablation sizes obtained by experimental measurement, numerical simulation and the method proposed here. The group column in the table indicates the pulse parameters shown in Table 1. Every pulse protocol was repeated three times and the experimental results were shown as mean ± standard deviation; the average value was used to calculate the errors of size acquired from simulation and the functions. The electric field thresholds (*E*_th_) for different pulse numbers were obtained by overlying the electric field contours on the real ablation zone to get a better match. *E*_th_ for 30 and 60 pulses was 180 V/cm while the value was 140 V/cm for 90 pulses. ImageJ was used to measure the length from different directions in the contours to get *X*_2_, *X*_3_, *X*_4_ and *X*_5_ shown in Fig. 2b. The results show that both the numerical simulation and the method proposed here can predict the ablation size. However, for numerical simulation, the exact *E*_th_ is unknown before treatment while the methods proposed here are based on real time impedance changes, so the results do not depend on *E*_th_.

**Table 2:** Ablation size acquired by different methods

Group	Experiment measurement (cm)	E_th_ for simulation (V/cm)	Size by simulation (cm)	Errors (%)	*R*_rel_	Size by functions (cm)	Errors (%)
10	X2 = 0.67 ± 0.05	180	0.608	8.57	12.12	0.72	7.49
10	X3 = 0.90 ± 0.11	180	1.126	25.2	2.71	1.02	13.90
	X4 = 0.96 ± 0.23		1.126	16.9	2.43	0.94	1.57
10	X5 = 1.49 ± 0.23	180	1.517	1.65	1.42	1.38	7.58
11	X2 = 0.70 ± 0.12	180	0.608	12.52	12.13	0.71	2.87
11	X3 = 1.23 ± 0.16	180	1.126	8.38	2.91	1.08	12.08
	X4 = 1.14 ± 0.11		1.126	0.94	2.72	1.03	9.47
11	X5 = 1.73 ± 0.10	180	1.517	12.46	1.50	1.43	17.4
12	X2 = 0.74 ± 0.15	140	0.734	0.94	13.63	0.77	4.50
12	X3 = 1.37 ± 0.02	140	1.359	1.09	3.92	1.36	0.98
	X4 = 1.35 ± 0.11		1.359	0.37	3.70	1.30	3.95
12	X5 = 1.74 ± 0.04	140	1.901	9.48	2.04	1.76	1.42

## Discussion

### Variation of the impedance spectrum

The double layer at the interface between the electrodes and potato tissue mainly affects the impedance at lower frequencies [19] and decreases exponentially with frequency (Fig. 3a). The cell membrane is usually equivalent to a capacitor in parallel with a resistor with a large resistance value. At low frequencies, the cell membrane has very high impedance and the capacitor is considered an open circuit. However, high frequencies the reactance of the capacitor is reduced and the impedance of the circuit model (Fig. 1b) is mainly determined by the equivalent extra-cellular and intra-cellular resistance. *R*_e_ and *R*_c_ are in parallel and *R*_c_ is much smaller than *R*_e_, so the total impedance is governed by *R*_c_, which will not change at high frequency, and will not be affected by microsecond pulses (Fig. 3a).

The change of the impedance spectrum, before and after treatment, is reflected by the change of the equivalent extracellular resistance defined in Eq. (2). The larger the *R*_rel_, the higher the degree of electroporation between the respective electrode pair. For 30, 500 V pulses, there is no change in impedance (*R*_rel_ = 1) from 3–4, meaning that the ablation zone was separated around electrodes 1 and 2 and not connected. This result was also verified by the experimental results (Fig. 2a).

The results in Fig. 3 show that the change of *R*_rel_ from electrode pair 1–2 has less statistical difference among different groups than other electrode pairs, especially for higher electric field strengths. There may be some reasons leading to this result: first, the chemical reactions, like electrolysis, in the vicinity of the electrodes when we applied high voltage pulses are difficult to estimate. Interestingly, this electrochemical process has been employed to enhance the ablation results by some groups [25]. However, the induced movement and the transfer of the ions introduce noise to the measured results [26]. Also, there may be a saturation of the measured results obtained from only two treatment electrodes when the ablation size is relatively large [27]. For two-electrode systems, the tissue around the electrodes contributes to the measured impedance and the further the tissue, the less the tissue contributes to the total impedance. The contribution weight is described by the impedance sensitivity [28, 29]. When the ablation size is relatively large, the tissue that is relatively far away from the electrodes contributes little to the total impedance, leading to saturation of the impedance variation. This is why when the ablation size is relatively large, the change in conductivity caused by electroporation will not induce a significant change in impedance of the tissue. Therefore, using different electrode pairs to measure the change in the impedance will increase the accuracy of the results. The last reason is that tissue is not absolutely homogenous and the inserting of the electrodes is not precise. This affects the impedance between all electrode pairs. Nowadays, the electrodes are all inserted manually, which makes it difficult to ensure that the relative position of the electrodes will not change during the insertion process. A more intelligent electrode setting method needs to be proposed in the future.

### Correlation of the ablation outcome and the impedance spectrum

The relationships between *k*_2_–*k*_5_ and the *R*_rel_ from respective electrode pair can be well described by linear functions. There is a step from 0 to the next value for *k*_5_; therefore, the linear relationship between *k*_5_ and *R*_rel_ from 3–4 does not include the first point, which is close to zero. The value of *k*_1_ will be the same when the ablation zone is connected. Thus, we do not use a function to fit these data. However, the relationships between *R*_rel_ from other electrode pairs and the relative positions (*k*_2_, *k*_3_, *k*_4_) can be well described by linear functions. To minimize the variability used to locate the ablation zone, we merge *k*_3_ and *k*_4_ into one parameter, *k*_3,4_. Normally, for IRE for tissue ablation, the electrical dosage is high enough to make the ablation zone connected. Therefore, we recommend *k*_2_, *k*_3,4_ and *k*_5_ as the main variables to describe the ablation zone. According to these functions, one can estimate the ablation size if *R*_rel_ can be calculated. The impedance of the tissue is determined by both the conductivity and the shape factor of the measurement system. The conductivity of the tissue is non-uniform, especially after electroporation. The corresponding shape factor for heterogeneous conductivity distributions is complicated. Unfortunately, there is no good way to measure the real conductivity distribution after electroporation. Therefore, in this study, we use the relative change of the impedance to quantify the variation of the tissue property. IRE often employs more than two electrodes to treat tumors; however, only two electrodes are used to apply pulses during one treatment while the other electrodes can be used to measure impedance. Therefore, the method proposed in this study can be transferred into different electrode configurations.

When compared to the numerical simulation, the method proposed in this study also can reflect the ablation size well. Due to the insufficient study on dynamic conductivity and the electric field threshold of IRE, the prediction of the ablation outcome by simulation for different pulse protocols needs to be calibrated by redefining the electric field threshold or including the temperature module. However, the method proposed here only depends on the change of the impedance in real time. It is quite simple and convenient to assess the ablation size by this method; however the drawback of this method is that it cannot give the image of the ablation but only some lengths in specific directions. Moreover, this method is based on the variation of the impedance; therefore, it cannot be conducted before treatment while numerical simulation is usually used to determine the pulse protocols before treatment which is an important part of treatment planning. The method proposed here, which can be used to monitoring the ablation outcome during the treatment, would be a significant supplement to the numerical simulation.

Traditionally, IRE treatments are delivered using a 4-electrode setup with only two electrodes being implemented at a given time (i.e., one source, one ground, two floating). After the first set of pulses are delivered, the energized electrodes are alternated and another set of pulses are delivered until all 2-electrode combinations have been delivered [30]. We believe that the method presented here can be readily used after the first set of pulses is delivered but future work is needed to assess if these finding could be expanded for a full treatment. There are several challenges including eletrochemical and electroporation hysteresis effects that would need to be accounted for clinical adoption. Effects from prior electroporation would be needed to be eventually studied as it would be impractical for clinicians to add more electrodes than what is needed to deliver the therapy. We do believe that eventually this 4-electrode approach, which is more commonly used for bioimpedance analysis that a 2-electrode approach, could be a more robust way to monitor treatment outcome if these effects are appropriately captured.

In this study, the impedance spectrum from 1 Hz–1 MHz was measured. It takes about 2 min to finish measuring all four electrode pairs after treatment and connecting the electrodes and the impedance measurement equipment after treatment was done manually. All of these need to be improved in the future because the impedance after IRE has a large temporal dependence. In practical applications, only lower frequency information is needed to quantify the impedance change, making it possible to measure the impedance during treatment [31, 32]. Another option is to use a test pulse during treatment, then converting the recorded voltage and current into the frequency domain by Fourier transform to calculate the impedance [33].

The main limitation of this method is that it is empirical and these relationships cannot be extended to a wider range of pulse parameters. This is why *R*_rel_ = 1 (no change of the measured impedance) and the equation given in the Fig. 4 gives a positive value while it should be 0. It is typically based on the experimental phenomena and it shows that in a certain pulse parameter range, the ablation zone could be reflected by the change of the tissue impedance according to a specific function. However, the physical mechanism should be further studied. Currently, it is only tested in the relatively homogeneous potato tissue to support the method. Experimental confirmation on different heterogeneous animal tissues, electrode configurations and wider pulse parameters are needed to validate the method.

## Conclusions

In this paper, we utilized a four-electrode configuration using a tuber model to investigate the impedance change before and after treatment. The change in tissue impedance measured from the non-pulsing electrode pair had lower deviations compared to the pulsing electrode pair, suggesting that electrodes can be used to reliably detect impedance changes in IRE tissue ablation. The relationship between the impedance change and the ablation size was determined by linear fitting, which was beneficial in ablation zone estimation.

## Abbreviations

PEFs: pulsed electric fields
IRE: irreversible electroporation
EIT: electrical impedance tomography
MREIT: magnetic resonance electrical impedance tomography
ECM: equivalent circuit model
CPE: constant phase element
